# The association between intolerance of uncertainty and psychological burden among caregivers of children with autism and the impact on their quality of life

**DOI:** 10.3389/fpsyt.2025.1492304

**Published:** 2025-04-25

**Authors:** Khulood Mohammed Al Mansoor

**Affiliations:** Self-Development Skills Department, King Saud University, Riyadh, Saudi Arabia

**Keywords:** intolerance of uncertainty, burden, quality of life, caregivers, autism

## Abstract

**Introduction:**

Caregivers of children with Autism Spectrum Disorder (ASD) often face significant stressors, including financial strain, social stigma, emotional exhaustion, and unpredictable daily routines. These challenges can severely impact their quality of life (QoL). This study aimed to examine the relationship between intolerance of uncertainty, caregiver burden, and QoL among caregivers of children with autism.

**Methods:**

A cross-sectional study was conducted with 59 caregivers from six branches of the Obour Company for Human Development in Riyadh. Data were collected electronically using a sociodemographic data sheet, the Intolerance of Uncertainty Scale, the Zarit Burden Interview (short form), and the World Health Organization Quality of Life Scale (brief form).

**Results:**

Findings revealed that two-thirds of caregivers experienced high to moderate levels of intolerance to uncertainty and a moderate burden, while 13.6% reported a high burden. Nearly 60% of participants reported low overall QoL, particularly in the psychological and social domains. A significant positive correlation was found between intolerance of uncertainty and caregiver burden. Additionally, significant negative associations were observed between QoL scores and both intolerance of uncertainty and caregiver burden, except in the environmental domain. Intolerance of uncertainty emerged as a significant inverse predictor of overall QoL.

**Discussion:**

These results emphasize the psychological toll of caregiving for children with ASD. Interventions such as family- and community-based support programs and child behavioral training are essential to reduce caregiver burden and enhance QoL. Tailored services should be prioritized in clinical practice to support caregivers more effectively.

## Introduction

Autism spectrum disorder, commonly referred to as ASD, is a neurodevelopmental disorder marked by unique patterns in social communication and a persistent, varied nature (1) associated with constrained, monotonous, or stereotypical behaviors (2). All patients with ASD show deficits in verbal and nonverbal social communication and cognitive and motor skills and often have unusual hobbies, repetitive actions, and atypical reactions to sensory stimuli (3). The numerous outward manifestations of ASD include sleep issues, hostility, self-injury, hyperactivity, impulsiveness, and abnormally weak or strong sensitivity to stimuli. The problematic behaviors of children with ASD present significant difficulties for their parents or other caregivers, who experience stress and challenges related to their work, finances, estrangement, unpredictable lives, and social stigma. In addition to physical and mental fatigue, these factors significantly lower their families’ quality of life (QoL) (4, 5). ASD prevalence has increased globally over the past three decades (6, 7). Recent epidemiological studies have revealed a sharp rise in ASD prevalence, with boys 4–5 times more likely than girls to have ASD. It has been estimated that 1% of individuals in North America, Europe, and Asia have ASD (8, 9), while the frequency of ASD is estimated to be 3.9% in Asia and 0.14%–2.9% in the Arab nations around the Persian Gulf (10, 11).

The most recent definition of intolerance of uncertainty (IU) is “an individual’s dispositional inability to endure the adverse response caused by the perceived absence of salient, crucial, or sufficient information, and perpetuated by the related feeling of uncertainty” (12). Therefore, when an individual encounters a stimulus they fear, like the unknown, they experience a fear reaction perpetuated by their views of uncertainty as unpleasant (i.e., IU). The tendency for an individual to feel afraid due to an apparent lack of information at any level of consciousness or processing is known as fear of the unknown (13). IU is an important transdiagnostic process associated with anxiety (12). Individuals with IU tend characteristically to have negative emotional, cognitive, and behavioral responses when encountering unknown situations (14). According to Bailey et al. (15), uncertainty develops “when a person in a decision-making position is unable to assign a value to the objects and events and/or when the person fails to predict the consequences owing to a lack of sufficient information.” As previously noted, generalized anxiety disorder has been associated with IU (16) and major depressive disorder (17). Furthermore, in two groups of parents of children with ASD, it was discovered that a higher intolerance for ambiguity predicted higher levels of negative affective symptoms, such as stress, anxiety, and depression (18).

Caregiver burden is the degree to which those providing care feel that their physical and emotional well-being, social lives, and finances are suffering due to a family member’s illness (19, 20). The related term “care burden” refers to a collection of psychological, emotional, social, and financial difficulties that an individual caring for someone with a physical or mental illness must overcome. These difficulties can result in psychological issues, poor QoL, depleted energy, fatigue, and physical dysfunction (21). A distinction is frequently made in the literature between caregivers’ objective burden, meaning actual problems (such as strained family relationships; limitations on social, leisure, and employment activities; and money problems), and their subjective burden, meaning their psychological reactions, such as dissatisfaction with their abilities, depression, anxiety, and social embarrassment (22). Therefore, caring for children with autism is challenging and tiresome, requiring an organized program and considerable effort and time (23). Just as ASD has a wide range of direct effects, such effects are also felt by parents and families of children with ASD, who must invest significant time, effort, and patience to meet the demanding care needs of affected children. ASD is severe and persistent. Children with autism have numerous medical and developmental comorbidities, and health services find it difficult to provide the integrated and intensive interventions that patients with ASD require. These factors make it challenging to care for children with ASD (24). The heavy burden of caring for a child with ASD is reflected in how broad and severe the areas of parenthood that appear to be affected are (25). Caregiving may be stressful, challenging, and demanding, affecting family members’ physical and mental health (26–28).

The emotional responses encompass feelings of anxiousness owing to ambiguity, grief and guilt resulting from loss, and wrath deriving from difficulties in accepting the circumstance. These feelings might have a role in the emergence of mental and physical health issues (29). These distressing circumstances highlight the need for support networks that comprehend the multifaceted difficulties encountered by carers of children with autism spectrum disorder. (30).

QoL denotes the well-being and satisfaction an individual expects from their social and cultural achievements. It is a multifaceted, subjective construct that includes cognitive (satisfaction) and emotional (happiness) components in addition to physical, emotional, and social domains (31). QoL is a potentially helpful indicator of parents’ adaptation to their child’s ASD and is a crucial step in fully comprehending the struggles and experiences of individuals with ASD and their families (32, 33), since long-lasting and multifaceted clinical concerns related to syndromic ASDs are connected to adverse lifelong health and socioeconomic effects for children and their caregivers (34, 35). Some difficulties that impair parents’ psychological well-being are financial issues, a lack of social support, poor access to healthcare, and being diagnosed with autism (10, 36). The need for frequent medical consultations and treatments, specialized schooling, and coordinated family assistance places an increased financial burden on those caring for a child with ASD (37). Consequently, parents may decide to change employers, forgo seeking work, or quit their current employment to better meet their child’s requirements (38). Caretakers of children with ASD are more susceptible to a poor QoL due to the dynamic nature of the obstacles they confront. Caregivers must learn to accept, adjust, and deal with new knowledge and requirements when an ASD diagnosis is first given (39). Their challenges increase as the child approaches the ages of four to eight years due to additional comorbidities and intensified emotional or behavioral symptoms (40).

### Study significance

The biggest challenges with children with autism are generally caused by external, systemic factors such as limited access to health care, education, and community-based support services (41, 42). Care for children with impairments such as autism is different and not due to the characteristics of the children themselves but due to external and institutional barriers that fail to offer adequate support to families. Such challenges are time-consuming, and require more care and financial resources due to special needs concerning support. When there is no adequate systemic support, a single parent or other caretaker is left with too much to do and is more susceptible to depression and other psychosocial difficulties (43). All family members are hence impacted by having a child with autism, although primary caretakers are found to be more psychologically vulnerable.

In place of deficit or burden perspectives on autistic children, it is important to see them as individuals and to consider how structural shortcomings cause difficulties for carers. Parental stress is more likely to arise through coping with *the* inaccessibility of services, lack of clarity about resources available to them, and *the* absence of inclusive structures in society. Identification of these external stressors is more consistent with a social model of disability and allows for a strength-based conception of autism. This study is a step towards a more advanced understanding by considering how intolerance of *uncertainty and burden on caregivers*—both factors deeply entrenched in external and systemic factors—affect quality of life. To our knowledge, there has been virtually no previous study of associations between the variables investigated by this study. This study bridges a crucial gap by quantifying *the* association between IU and psychological burden reported by *caregivers of* children with autism and impact on QoL and calling for more inclusive support structures that promote *caregivers* welfare and celebrate neurodiversity.”

### Study aim

This study aims to examine the association between IU and psychological burden among caregivers of children with autism and how these factors affect caregivers’ QoL.

### Research hypotheses

H1: There is an association between IU and psychological burden.

H2: QoL is compromised by caregivers’ IU and psychological burden.

## Materials and methods

### Research design and setting

A web-based survey was used to collect cross-sectional data from a convenience sample of caregivers of children with autism. This study was conducted at the six Riyadh branches of the Obour Company for Human Development, whose 22 branches across Saudi Arabia deliver services, including several types of psychotherapy, communication therapy, speech therapy, and rehabilitation, to individuals with ASD.

### Sample

The convenience sample comprised 59 caregivers of children with autism who attended the study settings. The inclusion criteria were caregivers who agreed to participate, aged >18 years, able to read and write Arabic, and the primary caregiver of a child with ASD for at least one year and under treatment in the study setting. The sample of 59 caregivers was sufficiently large to demonstrate a correlation coefficient equal to −0.47 (44) with a statistical power of 95%, a confidence level of 95%, and a dropout rate of 10% (45).

### Data collection tools

The self-administered questionnaire included items to assess scores on the Intolerance of Uncertainty Scale (IUS), the 12-item short form of the Zarit Burden Interview (12-ZBI), and the brief form of the World Health Organization Quality of Life scale (WHOQOL-BREF). The sociodemographic and clinical data sheet collected information on participating caregivers’ sex, age, marital status, education, occupation, monthly income, whether they had a chronic condition, whether they had children with ASD, and other personal details, including the length of the child’s autism diagnosis, whether more than one child in the family had autism, whether the child had any other disorders, whether they were receiving therapy for their condition, the type of treatment, and whether the caregiver was devoted to monitoring their child’s progress.

#### Tool I: the IUS

According to Freeston et al. (46), the 27-item IUS was initially developed in Quebec to measure how individuals react to ambiguous situations, uncertainty, and potential outcomes. Buhr and Dugas (47) (47) later created a verified English version of this scale. The IUS-12 (48) is a condensed version of the original IUS, comprising 12 items scored on a Likert-type scale from one (not at all like me) to five (entirely like me). These fall into two categories (48, 49) seven IU-prospective items about future occurrences (e.g., “I can’t bear being taken by surprise”) and five IU-inhibitory items about ambiguity, preventing action, or experience. Both variables have a strong internal consistency of 0.85 (48). Previous studies have shown that the IUS-12 correlates significantly with the original IUS (*r* = 0.94–0.96) and has a continuous latent structure (12, 48, 50).

#### Tool II: the 12-ZBI

Caregivers’ psychological and social burden was assessed by the 12-ZBI (51) developed from the original ZBI (52). Respondents were required to rate their level of agreement with statements on their feelings regarding the results of caring on a five-point scale from 0 (never) to 4 (nearly always), producing a final score that ranges from 0 to 48. Gratão et al., (53) found that the 12-ZBI had excellent internal consistency, with Cronbach’s alpha of 0.81.

#### Tool III: the WHOQOL-BREF scale

Developed by Skevington et al., (54), the WHOQOL-BREF is a short form of the WHOQOL Scale and comprises 26 questions. Two items ask about the participants’ general assessment of their QoL, and the remaining 24 correspond to the original scale’s four domains: physical, psychological, social, and environmental (31). Therefore, WHOQOL-BREF creates a profile based on two individually scored items and four domain scores about an individual’s overall opinion of their QoL and health. The scale for the four domain scores is positive, with higher scores suggesting better QoL. The English version was translated into Arabic (the local language), and translation specialists verified the accuracy of the Arabic version. QoL was assessed as poor for scores <50%, acceptable at 50%–75%, and good at >75%. A previous found its Cronbach’s alpha to be 0.737, indicating acceptable reliability in terms of internal consistency (55).

### Pilot study

The pilot study included 10% of the caregivers meeting the inclusion criteria. It tested the feasibility and clarity of the scales and determined the time required to complete the questionnaire. The Arabic translation of the questionnaire was subjected to reverse translation and then tested for validity and reliability. Its content validity was approved by five experts in child psychiatric medicine and a psychologist. No changes were found to be required, and the pilot data were not excluded from the primary study. The questionnaire used simple, self-explanatory language that enabled completion in ≤30 minutes. Test-retest reliability was detected by using Cronbach’s alpha test, which detected that all scales utilized in the present study have good reliability: IUS (α = 0.94), ZBI (α = 0.89), and WHOQOL-BREF (α = 0.98).

### Study procedure

Electronic invitations were sent to potential participants in the pilot and primary studies. The online survey, which took 25–30 minutes to complete, was built using Google Forms and sent to participants through WhatsApp, Twitter, and emails. Participants were given a debriefing after the survey, and the study did not use any deceit. Only non-personally identifying data were gathered. Data were collected over four months (December 2022 to March 2023) until the number of responses corresponded to the planned sample size.

### Statistical analysis

All data were collected, tabulated, and statistically analyzed using the IBM SPSS statistical package (version 23.0; IBM Corp., Armonk, NY, USA). Quantitative data are expressed as mean ± standard deviation (SD), median, and range. Qualitative data are expressed as absolute and relative frequency (number and percentage). Categorical variables were compared using the chi-squared test. The relationships between pairs of variables were assessed using Pearson’s product-moment correlation coefficient (*r*), where a + sign indicates a direct correlation and a − sign indicates an inverse correlation, with a value of 1 indicating the strongest correlation and 0 no correlation. All tests were two-sided. Statistical significance was set at *p* < 0.05. Regression coefficients (*β*) were also calculated, and the *R* ^2^ test was used to assess multiple linear regression which makes the use of many explanatory factors for predicting the value of a response variable (i.e., the relationship of a dependent continuous variable with one or more independent continuous variables).

## Results

**Table 1** shows that the 59 participating caregivers ranged in age from 18 to 64 years, with a median of 40 years. They were predominantly female (71.2%) and married (78%), with either a Bachelor’s degree or a diploma (66%). Slightly more than half (52.2%) did not work, and most had a low or insufficient monthly income (71.2%). Only one-fifth had a chronic illness (20.3%).

**Table 1:** Participants’ characteristics.

Variable	*n*	%
Sex		
Female	42	71.2
Male	17	28.8
Age	Mean ± SD: 39.56 ± 10.00 Median (range): 40 (18–64)	
<40 years	27	45.8
≥40 years	32	54.2
Marital status		
Married	46	78.0
Single	4	6.8
Divorced	7	11.9
Widowed	2	3.4
Education		
Basic	8	13.6
Diploma	14	23.7
Secondary	7	11.9
Bachelor’s	25	42.4
Postgraduate	5	8.5
Occupation		
Not working	31	52.5
Working	14	23.7
Housewife	14	23.7
Income		
Mid-level	17	28.8
Low-level	42	71.2
Chronic disease		
Yes	12	20.3
No	47	79.7

**Table 2** shows that approximately two-thirds of the caregivers had high to moderate intolerance for uncertainty (61%), 59.3% had a moderate burden, and 13.6% had a high burden. **Table 3** shows that more than half of caregivers (59.3%) had a generally low QoL, with remarkably low mean scores in the social (9.2 ± 2.9) and psychological (17.1 ± 5.5) WHOQOL-BREF domains. **Table 4** shows significant positive correlations among the IUS and burden scores. It also demonstrates statistically significant negative correlations between total QoL scores and all of its domains except the environmental domain and between IUS and burden total scores at p=0.01. **Table 5** indicates that the IUS score was a significant inverse predictor of QoL in caregivers. Otherwise, there were no significant parameters (p > 0.05).

**Table 2:** Intolerance of uncertainty and burden on caregivers of children with autism.

	Level*						Score	
	High		Moderate		Low		Mean ± SD	Median (range)
Intolerance of uncertainty	18	30.5	18	30.5	23	39.0	40.2 ± 10.3	41 (16–60)
Burden on caregivers	8	13.6	35	59.3	16	27.1	26.1 ± 10.2	27 (2–48)

*Scale: Low: <50%; moderate: 50%–75%; high: ≥75%.

(Approximately two-thirds of the caregivers had high to moderate intolerance for uncertainty (61%), 59.3% had a moderate burden, and 13.6% had a high burden).

**Table 3:** Quality of life of caregivers of children with autism.

How would you rate your quality of life?	Very poor		Poor		Neither poor nor good		Good	Very good
	6 (10.2)		8 (13.6)		30 (50.8)		10 (16.9)	5 (8.5)
How satisfied are you with your health?	Very dissatisfied		Dissatisfied		Neither dissatisfied nor satisfied		Satisfied	Very satisfied
	4 (6.8)		6 (10.2)		22 (37.3)		22 (37.3)	5 (8.5)

WHOQOL-BREF domain	QoL level						QoL score	
	Good		Fair		Low		Mean ± SD	Median (range)
	*n*	%	*n*	%	*n*	%		
Physical	8	13.6	24	40.7	27	45.8	21.4 ± 5.4	21 (11–35)
Psychological	8	13.6	16	27.1	35	59.3	17.1 ± 5.5	16 (8–29)
Social	11	18.6	27	45.8	21	35.6	9.2 ± 2.9	9 (3–15)
Environmental	10	16.9	17	28.8	32	54.2	23.3 ± 6.9	23 (8–38)
Total QoL	7	11.9	17	28.8	35	59.3	71.1 ± 18.9	69 (33–116)

QoL, quality of life.

(More than half of caregivers (59.3%) had a generally low QoL, with remarkably low mean scores in the social (9.2 ± 2.9) and psychological (17.1 ± 5.5) WHOQOL-BREF domains).

**Table 4:** Correlations between total intolerance of uncertainty, burden, QoL, physical, psychological, social, and environmental domains.

Variables	IUS score		Burden score		QoL score	
	*r*	*p*	*r*	*p*	*r*	*p*
Intolerance of uncertainty score	1.000					
Burden score	+0.488**	0.0001	1.000			
Total QoL score	−0.377**	0.0030	−0.593**	0.0001	1.000	
Physical domain score	−0.411**	0.0010	−0.592**	0.0001		
Psychological domain score	−0.369**	0.0040	−0.603**	0.0001		
Social domain score	−0.423**	0.0010	−0.569**	0.0001		
Environmental domain score	−0.229	0.0810	−0.428**	0.0010		
Age of caregivers	+0.030	0.822	+0.272*	0.0370	−0.205	0.1190
Child age (years)	−0.101	0.445	+0.099	0.4540	−0.176	0.1840
Disease duration	−0.040	0.763	+0.095	0.4760	−0.182	0.1720

*r*, Pearson’s product-moment correlation coefficient; *p < 0.05 (two-tailed); **p < 0.01 (two-tailed).

(**Table 4** shows significant positive correlations among the IUS and burden scores. It also demonstrates statistically significant negative correlations between total QoL scores and all of its domains except the environmental domain and between IUS and burden total scores at p=0.01).

**Table 5:** Multiple linear regression model for predicting QoL among caregivers.

Predictors	Unstandardized coefficients		*t*	Sig.	*r*	*R* ^2^
	β	SE				
(Constant)	105.5				0.640	0.410
Intolerance of uncertainty score	−0.261	0.230	1.13	0.2610		
Burden score	−0.965	0.237	4.10	0.0001*		
Chronic disease	−9.849	6.500	1.50	0.1360		

β, regression coefficient; SE, standard error; *R* ^2^ = 41% of predictors; *f*-test = 5.9; *p < 0.0001.

(**Table 5** indicates that the IUS score was a significant inverse predictor of QoL in caregivers. Otherwise, there were no significant parameters (*p* > 0.05)).

## Discussion

Living with a child with autism affects all family members, with primary caregivers considered at greater psychosocial risk. This study primarily investigated the association between IU and psychological burden among caregivers of children with autism and how these factors affected their QoL.

The participating caregivers were mainly female, aged around 40 years, married, and unemployed, with a bachelor’s degree or diploma. The high proportion of female caregivers is consistent with women’s traditional role of taking the major responsibility for caring for their ill relatives. Many older parents may also care for non-ASD family members, whether children, spouses, or parents, while having a child with ASD may make it problematic for a parent to pursue a career because of the prolonged caregiving requirements. Moreover, parents’ greater awareness that results in early referral and intervention can be attributed to their higher level of education.

These results are consistent with an earlier Chinese study (56), where the responding parents’ mean age was 39.07 ± 6.03 years, and about one-third had earned undergraduate or graduate degrees. Our findings are also consistent with a previous Malaysian study whose sample of parents predominantly held a degree or diploma/certificate, with less than 4% having completed only primary education (57). Similarly, a recent Saudi Arabian study found that participants were highly educated (58). Another study in Eastern Saudi Arabia reported that most parents of children with autism who participated were female, had a mean age of 45 years, were married, and held a Bachelor’s degree (59).

An Indian study in the same year included 70 participants, with most being female, which they noted was consistent with the perception of females as naturally more caring so that society encourages them to perform roles conventionally connected with nurture and caregiving. Worldwide, most individuals who care informally for relatives with long-term diseases or impairments, including the old and mentally ill, are women. Therefore, some claim that women are under pressure from society and culture to take on the role of family caregivers (60).

Our study has found that approximately two-thirds of participating caregivers had moderate to high intolerance for uncertainty, suggesting that having a child diagnosed with autism, in addition to its apparent behavioral, emotional, and psychological manifestations, may provoke feelings of intense anxiousness and uncertainty about the child’s future. Childhood diseases may also be perceived as novel, complex problems that are hard to address and are therefore seen as a danger, so thinking of them as immutable either reflects or ignores the reality of the situation. IU occurs when an individual is unable to handle this situation. This finding is consistent with a Turkish study that found parents of children with impairments were much more intolerant of uncertainty than parents of children without impairments (44). Other studies have similarly found that parents of children with ASD often show a high level of intolerance for uncertainty because they experience dread and anxiety due to the uncertainties and unknowns about their children (61–63). An Australian study also concluded that higher levels of IU and greater use of avoidant coping predicted anxiety and sadness in mothers of children with ASD (64).

Our study found that over half of participating caregivers perceived a moderate burden. There were significant positive correlations among the measures of IU and burden, providing support for our first hypothesis. This finding may be explained by the inability of their children with ASD to care for themselves and their consequent dependence on others, which are the two main problems with children with autism. The consequent care burden is one of the most challenging obstacles to the maintenance and care of such children; the parenting activities of their caregivers cause them to experience adverse physical and mental effects. Importantly, IU will tend to hamper an individual’s ability to perform tasks effectively, manifested when they no longer react appropriately regarding their perceptions and actions to sudden extraordinary circumstances. This inappropriateness could indicate a caregiver’s need for psychological support and education to improve their coping abilities and alleviate their negative emotions, uncertainty, and psychological burden. This conclusion is reinforced by a Jordanian study in which parents of children with autism reported moderate burdens (65). A Nepalese study (66) found that caring for children with ASD was perceived as a moderate to severe burden, consistent with our inference. Another earlier study found that in two independent groups of parents of children with ASD, higher levels of IU predicted higher levels of negative affective symptoms such as stress, depression, and anxiety (18). The burden appeared heavier in a recent Iranian study, which found that the mothers of children with ASD reported high levels of caring burden. The difference in results may be attributable to the sample’s nature since that study focused only on the mothers and was conducted during the COVID-19 pandemic, increasing the childcare burden (67).

Our findings demonstrate that most of the surveyed caregivers had a generally reduced QoL, their mean scores being notably lower in the social and psychological domains. This finding may reflect the majority of care being given by family members in the home, posing many difficulties for these families as they cover various caregiving responsibilities. For example, the child with ASD may need more social, psychological, and physical support than parents of children without ASD may routinely provide. Consequently, caregiving occupies a great deal of parents’ time, potentially limiting their ability to socialize and perform daily routines, so they neglect their own physical health and risk psychological effects, including anxiety, loneliness, and depression. This finding is consistent with a previous Saudi Arabian study that reported that most participating parents had a compromised QoL (68). Similarly, another Saudi Arabian study reported that parents caring for children with ASDs had lower scores than caregivers of other types in most QoL domains (69).

Our conclusion is reinforced by published qualitative findings showing that parents of patients with autism were socially discordant with their peers of the same age due to caring for adult offspring with ASD. Many parents found that their difficulties in interacting socially with peers of their own age were exacerbated by the ongoing and persistent need to provide care and assistance to their child with ASD into adulthood (34).

Our results show significant negative correlations of total QoL scores and scores in the physical, psychological, and social domains with the total scores for IU and burden. This finding supports our hypothesis that caregivers’ IU and burden impair their QoL. It demonstrates the impact that autism can have on caregivers and emphasizes the significance of considering this issue while providing therapy, which is consistent with earlier studies showing that parents of individuals with ASD bear heavy burdens (34) and that this frequently damages their psychological well-being and QoL (70). Similarly, a recent study found that parental energy and well-being were negatively impacted by uncertainty (71), while another study underlined the necessity of a family-centered strategy for dealing with the challenges caused by uncertainty (72). Indeed, recent advances in parent-mediated therapies for children with autism who struggle with uncertainty indicate promise in addressing how these challenges affect the daily lives of both the affected children and their parents (73, 74).

## Conclusions

Our study found that approximately two-thirds of participating caregivers had high to moderate intolerance for uncertainty and experienced moderate burden, with only 13.6% experiencing a heavy burden. Caregivers were found to have generally poor QoL, with particularly low mean scores in the social and psychological domains. Significant positive correlations existed between IUS scores and total burden scores, and significant negative correlations existed between total QoL scores and all QoL domain scores except the environmental domain and total IU and burden scores. Lastly, the IUS score was a significant inverse predictor of caregivers’ QoL. There were no other significant predictors.

### Study limitations

This study thoroughly explores the association between IU and the psychological burden perceived by caregivers of children with autism and the impact on their QoL, offering valuable insights. However, some limitations evolved about potential response bias from self-reported data and the inability to establish causality due to the cross-sectional design. Future research using longitudinal approaches and objective measures could provide deeper insights. Exploring other influencing factors may also enhance understanding, such as mental health status and socioeconomic, family dynamic, and cultural variables, particularly among caregivers of children with autism.

### Recommendations

I argue that healthcare practitioners must be more aware of the high-risk QoL impairment experienced by the parents of children with autism. There is a need to design and make available programs, such as family- and community-based services and child behavioral training, to caregivers of children with autism in clinical practice. Parents’ IU and burden may be reduced, their QoL improved, and positive outcomes promoted by strengthening systemic collaboration in providing healthcare, education, and social well-being services.

## Data Availability

The original contributions presented in the study are included in the article/supplementary material. Further inquiries can be directed to the corresponding author.
